# The impact of multimorbidity on the occurrence of depression among middle-aged and elderly people in China

**DOI:** 10.1371/journal.pone.0340673

**Published:** 2026-01-27

**Authors:** Biqi Zu, Peng Zhang, Ziwei Xie, Jinsong Huang, Yunan Zhang, Meiling Tang, Yulan Wu, Lijun Fan

**Affiliations:** 1 Department of Psychiatry, Dalian Seventh People’s Hospital, Dalian, Liaoning, China; 2 Department of Teaching and Research, School of Nursing, Dalian University, Dalian, Liaoning, China; 3 Department of Teaching and Research, School of Nursing, Qiqihar Medical College, Qiqihar, Heilongjiang, China; University of Padua: Universita degli Studi di Padova, ITALY

## Abstract

**Objective:**

This study aims to investigate the association between multimorbidity and the incidence of depression in the middle-aged and elderly populations in China, focusing on the impact of multiple chronic diseases on mental health outcomes.

**Methods:**

This research comprises a rigorous retrospective cohort study utilizing the esteemed China Health and Retirement Longitudinal Study (CHARLS) data. Follow-up data were meticulously analyzed from 2013 (Wave 2) to 2020 (Wave 5), with data from 2015 serving as the essential baseline for examining multimorbidity and depression. We employed Directed Acyclic Graphs (DAGs) to identify potential confounding factors, applying overlap weighting to effectively mitigate their influence, thus ensuring that intergroup comparisons mirror the integrity of a randomized trial. The association between multimorbidity and depression was systematically evaluated through Cox regression models, supplemented by thorough subgroup analyses and sensitivity analyses to confirm the robustness of our findings.

**Results:**

Our analysis included 3,495 participants aged 45 and older. Multimorbidity was found to significantly elevate the risk of depression (Hazard Ratio [HR] = 1.431, 95% Confidence Interval [CI] 1.202–1.703). This risk was particularly pronounced in women (HR = 1.617, 95% CI: 1.290–2.027) and older adults (HR = 1.482, 95% CI: 1.221–1.799). The risk of depression increased with the number of chronic diseases, particularly with two (HR = 1.423, 95% CI: 1.192–1.701) or three (HR = 2.045, 95% CI: 1.503–2.784) chronic conditions. However, this association diminished in significance when four chronic conditions were present (HR = 0.815, 95% CI: 0.444–1.497).

**Conclusion:**

The pronounced association between multimorbidity and depression in middle-aged and elderly individuals highlights an urgent public health issue, particularly for women and older adults. This study’s use of overlap weighting provides strong evidence of the substantial impact of multimorbidity on mental health. It underscores the immediate need for targeted interventions to improve mental well-being in these vulnerable groups.

## 1. Introduction

Globally, the prevalence of chronic diseases is continuously increasing, particularly notable among the middle-aged and elderly populations [1]. As the population ages, the coexistence of multiple chronic diseases is becoming increasingly common, severely impacting the quality of life, physical function, and healthcare burden of middle-aged and elderly individuals [2]. Additionally, multimorbidity poses a significant threat to mental health, with depression regarded as one of the most severe mental disorders in the realm of psychological health [3]. Existing studies indicate that the complexity of daily living and the demands of self-management increase due to multimorbidity, leading to the accumulation of negative emotions and psychological stress, making it more likely to develop depressive symptoms compared to individuals with a single chronic disease. Furthermore, the physical functional decline and social isolation trends caused by chronic diseases further exacerbate the occurrence of depressive symptoms [4]. The worsening of depressive symptoms not only affects quality of life but is also closely associated with an increased risk of suicide, posing a substantial threat to the psychological health and safety of individuals [5]. In China, multimorbidity is equally prevalent, presenting a significant challenge to the physical and mental health of the elderly population [6,7].

Currently, scholars have extensively researched the issue of depression among middle-aged and elderly individuals with multimorbidity in China; however, most studies focus on exploring influencing factors and differ in their approaches to managing confounding variables, resulting in effect sizes that may not accurately reflect the genuine relationship between multimorbidity and the incidence of depression. Furthermore, most existing studies are based on cross-sectional data analysis and lack longitudinal research capable of reflecting changes in depressive symptoms. This study utilizes data from the China Health and Retirement Longitudinal Study (CHARLS). It employs a longitudinal cohort study design, using overlap weighting to reduce the influence of confounding factors, thereby simulating the random allocation method found in randomized controlled trials to minimize intergroup differences. This research aims to deeply explore the impact of multimorbidity on the incidence of depression among middle-aged and elderly individuals, providing empirical evidence to improve the mental health of this population.

## 2. Materials and methods

### 2.1 Data source

The China Health and Retirement Longitudinal Study (CHARLS) is a tracking survey of the health and retirement status of a representative population aged 45 and older in mainland China. It has collected follow-up data from 2011 to 2020. It is the first nationally representative survey of the middle-aged and elderly population in China, serving as a high-quality public micro-database nationally. This study utilizes data from CHARLS collected between 2013 (Wave 2) and 2020 (Wave 5). During the survey, each respondent who agreed to participate signed an informed consent form.

### 2.2 Study cohort and inclusion criteria

**Fig 1:**
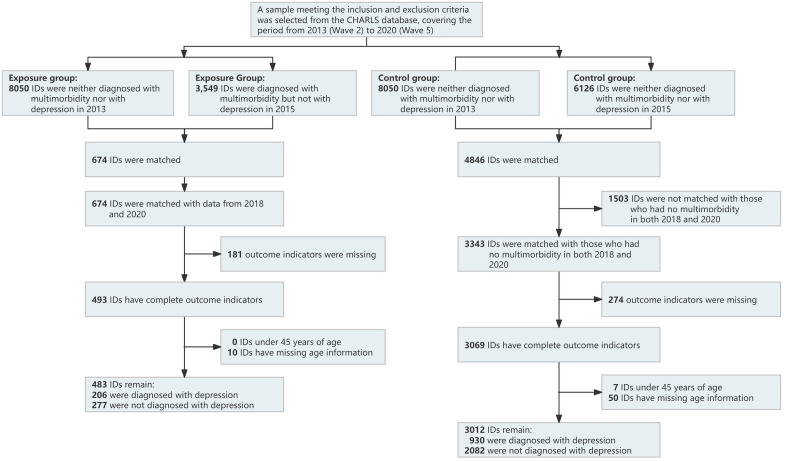
Data cleaning flowchart. This flowchart illustrates the steps taken to select and clean data for individuals from the CHARLS database across different years. Exposure Group (left): Participants diagnosed with multimorbidity and depression. Control Group (right): Participants neither diagnosed with multimorbidity nor depression. Matched IDs: Indicates successful matches across analysis waves. Remaining IDs: Final participant counts, indicating their diagnosis status.

This is a retrospective cohort study, with data collection beginning in 2013 (Wave 2). The research uses data from 2015 (Wave 3) as the baseline. It collects follow-up data from samples at the end of 2018 (Wave 4) or 2020 (Wave 5) to compare the impact of multimorbidity on the incidence of depression. This study selected samples aged ≥45 with a chronic disease count of ≥2, excluding samples with missing outcome indicators and those that failed ID matching over the years. To clarify the timing of depression onset after being diagnosed with multimorbidity, the study selected samples that were not diagnosed with multimorbidity or depression in 2013 but were diagnosed with multimorbidity and not diagnosed with depression in 2015 as the exposed group. The control group consisted of samples that were neither diagnosed with multimorbidity nor experiencing depression in both 2013 and 2015. Their data were observed during two subsequent follow-ups, specifically in 2018 (Wave 4) and 2020 (Wave 5). In order to exclude the impact of multimorbidity on depression in the control group, researchers required participants to be free of multimorbidity diagnoses in both 2018 and 2020. The data-cleaning process is illustrated in Fig 1.

### 2.3 Follow-up cut-off time

The follow-up is cut off when a sample is diagnosed with depression, which may occur in either 2018 (Wave 4) or 2020 (Wave 5); for samples not diagnosed with depression, the follow-up continues until it stops in 2020 (Wave 5).

### 2.4 Relevant definitions

#### 2.4.1 Outcome indicator: Depression.

Depression symptoms were screened using the 10-item Center for Epidemiological Studies Depression Scale (CESD-10) [8] based on participants’ status over the past week (feelings and behaviors from the previous week) in the CHARLS questionnaire. The scale includes ten items (eight negative and two positive). The negative items include: “I worry about little things,” “I have trouble concentrating while doing things,” “I feel sad,” “I feel that everything takes effort,” “I feel afraid,” “My sleep is restless,” “I feel lonely,” and “I feel I cannot go on with my life.” The positive items include: “I have hope for the future” and “I feel good.” Each item has four response options: “rarely or none of the time,” “not very much,” “some of the time or about half the time,” and “most of the time,” which are assigned scores from 0 to 3 (the positive items are reverse scored). The total score ranges from 0 to 30, with higher scores indicating more severe depression symptoms. In this study, depression symptom screening is classified into negative and positive categories, with scores below ten considered harmful and scores of ten or above considered positive for depression symptoms. The CESD-10 has demonstrated good reliability and validity, with a Cronbach’s α of 0.78, effectively screening for depression symptoms in the elderly population [9].

#### 2.4.2 Multimorbidity.

Based on responses to the CHARLS questionnaire item “Has a doctor ever diagnosed you with any of the following chronic diseases?” the types of chronic diseases include: hypertension, dyslipidemia (hyperlipidemia or hypolipidemia), diabetes or elevated blood sugar (including abnormal glucose tolerance or elevated fasting blood sugar), cancers (excluding mild skin cancers), chronic lung diseases (such as chronic bronchitis or emphysema), cor pulmonale (excluding tumors or cancer), liver diseases (excluding fatty liver, tumors, or cancer), heart diseases (such as myocardial infarction, coronary heart disease, angina, congestive heart failure, and other heart conditions), stroke, kidney diseases (excluding tumors or cancer), gastrointestinal or digestive system diseases (excluding tumors or cancer), emotional and mental issues, memory-related diseases (such as dementia or cerebral atrophy), Parkinson’s disease, arthritis or rheumatic diseases, and asthma (non-pulmonary diseases), totaling 14 types. Diagnosed with two or more chronic diseases is defined as “multimorbidity.”

#### 2.4.3 Hukou.

Hukou Type refers to China’s household registration system, which classifies individuals as either urban or rural residents. This classification affects access to social welfare, healthcare insurance, and other public services, and may therefore influence health outcomes and mental health status.

### 2.5 Ethics statement

The data used in this study comes from the China Health and Retirement Longitudinal Study (CHARLS). The CHARLS study received ethics approval from the Biomedical Ethics Committee of Peking University (Approval No: IRB00001052–11015). All participants provided informed consent prior to data collection. The informed consent process included a detailed explanation of the study’s purpose, data usage, and potential risks. All participants provided written consent. Additionally, the relevant ethics review board required ensuring that participants fully understood the study content and agreed to the use of their data in an anonymized manner. This study did not involve minors; therefore, parental or guardian consent was not required. Since this study utilized an existing publicly available dataset, the ethics committee did not require additional ethical review for this research.

## 3. Statistical methods

### 3.1 Statistical description

Continuous measures were summarized as median (IQR) for all samples. Categorical measures were reported as frequency and percentage for crude and matched samples and only for the weighted samples.

### 3.2 Control for confounding factors

To minimize the effects of confounding factors, the analysis used doubly robust estimation combining overlap weighting and outcome regression to compare outcomes in the exposure and control groups. *Overlap weighting* is a propensity score method that attempts to mimic essential features of randomized clinical trials. Weights are assigned to each sample proportional to the probability of that sample belonging to the opposite treatment group. This results in including all available samples and the exact balance for the mean of all covariates included in the model. In simulations, Overlap weighting has also shown improved precision relative to other weighting options.

Initially, we included 13 potential variables based on prior literature (Gender [10], age [11], smoking status [12], drinking status [13,14], educational level [15], insurance type [16], marital status [17], household registration type (Hukou type) [18], social activity participation [19], as well as body mass index (BMI), sleep duration, Activities of Daily Living (ADL), and Instrumental Activities of Daily Living (IADL) [20]) identified five confounding factors using Directed Acyclic Graphs (DAGs) (S1 Fig). These five confounding factors (Age, BMI, Sleep Duration, Companion Status, and Hukou Type) were used for overlap weighting.

Next, the new weight values were incorporated into the Cox regression model along with the confounding factors from overlap weighting and other potential confounding variables (Gender, Insurance Type, Education Level, Smoking Status, Drinking Status, Social Activity Status, ADL and IADL) for confounding adjustment, to assess the impact of multimorbidity on the incidence of depression. Additionally, age (less than 60 years, 60 years or older), gender, and the number of chronic diseases (2, 3, ≥ 4) were set as different groups for subgroup analysis to explore the impact of multimorbidity status on the incidence of depression in different subgroups. Furthermore, the incidence rates of depression diagnosed after 3 and 5 years of multimorbidity were also calculated.

### 3.3 Missing variables and data handling

Data exhibited missing values, particularly for Hukou Type and Education Level, where missing data was approximately 20%, while other data had relatively fewer missing values (for details on missing data, see **Table 1**). Multiple imputation [21] methods were used to handle the missing data for indicators and other variables. Five imputed datasets were constructed based on the data in **Table 1**, and the most appropriate imputation method was selected based on the data type. For continuous data, the Predictive Mean Matching (PMM) method was employed for imputation, while a regression imputation model was used for categorical variables to ensure data integrity. The Rubin formula was applied to obtain model estimates and compare the corrected standard errors of the imputations [22]. The outlier was removed for existing outliers (BMI > 100), and the mode was used for imputation.

**Table 1:** Baseline characteristics of overlap weighting results.

	Unweighted			Weighted^l^		
	Multimorbidity	Non-Multimorbidity	Standardized Mean Difference^l^	Multimorbidity	Non-Multimorbidity	Standardized Mean Difference^i^
No. of samples	483	3012				
No. of multimorbidity						
≤ 1	0	3012				
2	346	0				
3	87	0				
≥ 4	50	0				
Follow-up time (years)	4.63 ± 0.78	4.48 ± 0.88				
***Demographics***						
Gender, No. (%) or %						
Female	217 (44.93)	1343 (44.59)	0.0034	43.95	44.67	−0.0073
Male	266 (55.07)		1636 (55.41)	56.05		55.33
Age, median (IQR), y^a^	64 (56 to 71)	62 (56 to 68)	0.1606	62 (52 to 68)	62 (55 to 69)	-0.0186^j^
BMI, median (IQR)^bK^	24.45 (22.06 to 26.91)	23.62 (21.59 to 25.79)	0.1950	24.01(21.37 to 26.03)	24.02 (21.46 to 26.16)	−0.0028^j^
Hukou type, No. (%) or %^K^						
Urban	333 (68.94)	2379 (78.98)	−0.0590	73.10	79.00	0.0049^j^
Rural	140 (28.99)	610 (20.26)	0.0604	26.30	20.20	−0.0060^j^
Others	10 (2.07)	23 (0.76)	0.0019	0.60	0.80	0.0011^j^
Insurance type, No. (%) or %^K^						
Yibao	97 (20.08)	464 (15.41)	0.0344	15.87	15.91	−0.0004
Urban resident medical insurance	45 (9.32)	148 (4.91)	0.0295	7.32	5.06	0.0227
Hezuo yiliao	322 (66.66)	2304 (76.49)	−0.0672	73.65	75.83	−0.0218
Urban & rural resident medical insurance	6 (1.24)	37 (1.24)	0.0001	1.16	1.22	−0.0006
Gongfei	5 (1.04)	29 (0.96)	0.0007	0.88	0.99	−0.0010
Medical aid	0 (0.00)	1 (0.03)	−0.0003	0.00	0.03	−0.0003
Private medical insurance purchased by work unit	0 (0.00)	6 (0.20)	−0.0020	0.00	0.19	−0.0019
Private medical insurance purchased by individual	4 (0.83)	6 (0.20)	0.0021	0.32	0.20	0.0012
Other Medical Insurance	4 (0.83)	17 (0.56)	0.0026	0.80	0.57	0.0022
Education level, No. (%) or %^K^						
Primary school and below	255 (52.80)	1745 (57.94)	0.0211	62.19	57.54	0.0464
Middle school	138 (28.57)	820 (27.22)	0.0197	27.61	27.36	0.0025
High school and above	90 (18.63)	447 (14.84)	−0.0407	10.20	15.10	−0.0489
Smoking status, No. (%) or %^c^						
Yes	122 (25.26)	998 (33.13)	−0.0788	26.04	32.96	−0.0693
No	361 (74.74)		2014 (66.87)	73.96		67.04
Drinking status, No. (%) or %^dK^						
More than once a month	139 (28.78)	979 (32.50)	−0.0372	30.39	32.48	−0.0209
Less than once a month	50 (10.35)	293 (9.73)	0.0062	10.04	9.76	0.0029
Don’t drink	294 (60.87)	1740 (57.77)	0.0310	59.57	57.76	0.0180
***Other factors***						
Sleep duration, h, median (IQR)	7 (6 to 8)	7 (6 to 8)	-0.0919	7 (6 to 8)	7 (6 to 8)	0.0198^j^
Activity status, No. (%) or %^e^						
Intact	483 (100)	3012 (100)	0	100	100	0
Impaired	0 (0)		0 (0)	0		0
Companion status, No. (%) or %^f^						
Yes	425 (87.99)	2638 (87.58)	0.0041	87.87	87.67	0.0020^j^
No	58 (12.01)		374 (12.42)	12.13		12.33
ADL, No. (%) or %^g^						
Intact	381 (78.88)	2716 (90.17)	−0.1129	78.72	90.16	−0.1144
Impaired	102 (21.12)		296 (9.83)	21.28		9.84
IADL, No. (%) or %^hK^						
Intact	398 (82.40)	2940 (97.60)	−0.2183	75.28	97.60	−0.2232
Impaired	88 (17.60)		72 (2.40)	24.72		2.40

^a^Age: In this study, age was calculated based on the data release date of 2015 (Wave 2), which is June 20, 2019.

^b^BMI: body mass index, calculated as weight in kilograms divided by height in meters squared.

^c^Smoking status: Assign the value “Yes” to samples where “Still Smoke or already Quit” is “Yes” or where “Ever Smoked” is “Yes” and “Still Smoke or already Quit” is NA. Classify all others as “No”.

^d^Drinking status: Classify “Drink More Than Once A Month” as “More Than Once A Month,” “Drink But Less Than Once A Month” as “Less Than Once A Month,” and “None of These” as “Don’t Drinking.”

^e^Activity status: In the 2015 (Wave 2) data, a total of 12 activities were mentioned. If a sample can perform any one of these activities Normally, their activity status is considered “Intact.”

^f^Companion status: This study refers to spousal/partner accompaniment, determine companion status based on marriage status. Classify “Married with Spouse Present” and “Cohabitated” as “Yes,” and classify all others as “No.”

^g^ADL: Activities of Daily Living; select the six items: dressing, bathing, eating, getting in and out of bed, toileting, and continence. If any of these six items has a score >1 (Cannot be completed independently), it is recorded as “Functional Impairment.” [24]

^h^IADL: Instrumental Activities of Daily Living; select the six items: housekeeping, cooking, shopping independently, making phone calls, taking medication, and managing finances. If any of these items has a score >1 (Cannot be completed independently), it is recorded as “Functional Impairment.” [24]

^i^Absolute value of the between-group difference in means or proportions (Exposure group and control group) divided by the pooled SMD.

^j^Overlap weighting provided precise balance for these confounding variables.

^k^There were missing data for this variable. Missing values were handled with multiple imputation.For drinking status, there were missing data for 4 samples; IADL, there were missing data for 26 samples; BMI, there were missing data for 504 samples; Education level, there were missing data for 798 samples; Insurance type, there were missing data for 451 samples; Hukou type, there were missing data for 795 samples.

^l^After overlap weighting, a single sample No longer represents a single data entity and thus raw counts are Not reported after overlap weighting.

### 3.4 Sensitivity analysis

Propensity Score Matching (PSM) methods were utilized for sensitivity analysis, employing nearest neighbor matching with a caliper value set at 0.2 further to validate the effect size from the Cox regression. Additionally, to assess the robustness of the established association between multimorbidity and the incidence of depression against potential unmeasured confounding factors, the E-value was calculated using the method proposed by VanderWeele and Ding [23].

A significance level of 0.05 for two-sided comparisons was considered statistically significant. Hazard ratios (HRs) with 95% confidence intervals (CIs) are reported. Due to the nature of the study and the potential for type I errors resulting from multiple comparisons, all findings should be interpreted as exploratory. All analyses were conducted using RStudio version 4.3.3.

## 4. Results

### 4.1 Covariate overlap weighting results

**Fig 2:**
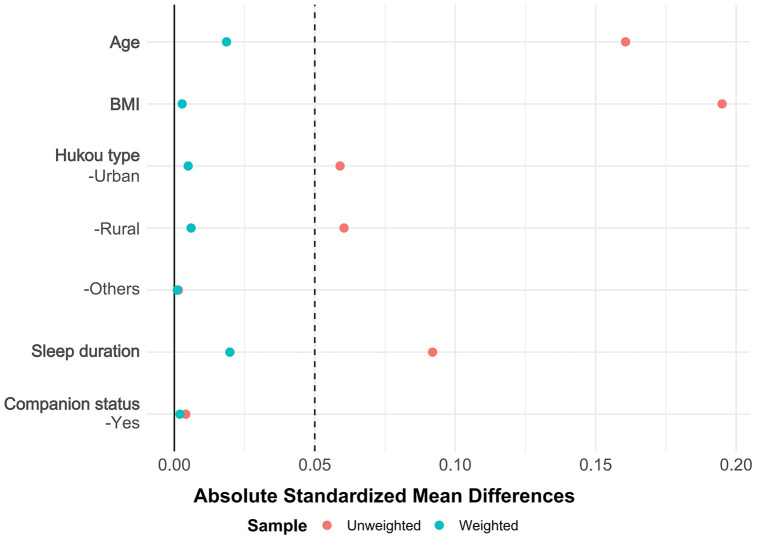
Results of Overlap Weighting for Confounding Factors. This figure displays the absolute standardized mean differences for Confounding Factors before and after overlap weighting. Red dots represent the unweighted sample, while blue dots indicate the weighted sample.

**Fig 3:**
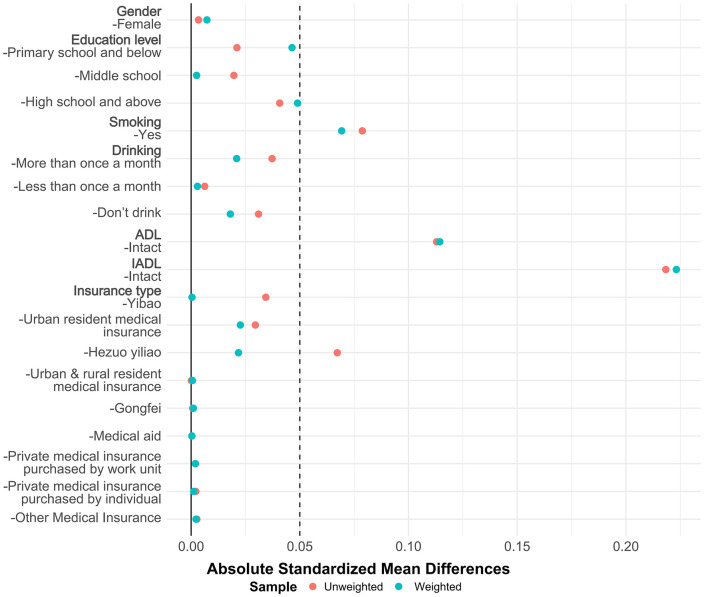
Results of Overlap Weighting for Potential Confounding Factors. This figure displays the absolute standardized mean differences for Potential Confounding Factors before and after overlap weighting. Red dots represent the unweighted sample, while blue dots indicate the weighted sample. Note: The variable “Activity status” remained “intact” in both groups and was therefore excluded from the overlap weighting procedure.

Based on the presence of multimorbidity, overlap weighting was applied to control for confounding factors in **Fig 2** and other potential confounders in **Fig 3** to ensure comparability between groups.

### 4.2 General results

This study included a total of 3,495 samples (1,560 females, accounting for 44.64%; median age: 62 years [IQR, 56–68 years]; median BMI: 23.70 [IQR, 21.64–25.87]; median sleep duration: 7 hours [IQR, 6–8]; 87.64% had a spouse/partner present; for Hukou type, 78.16% were urban, 21.08% were rural, and 0.76% were others).

After applying overlap weighting on the five variables—age, BMI, sleep duration, cohabitation with a spouse/partner, and Hukou type—the maximum balance between groups was achieved at baseline (see **Table 1**, Figs 2 and 3), with more significant changes in the Absolute Mean Differences values of the confounding factors between groups, in the sample after overlap weighting, 44.56% were female; median age: 62 years [IQR, 54–68 years]; median BMI: 24.01 [IQR, 21.42–26.14]; median sleep duration: 7 hours [IQR, 6–8]; 87.69% had a spouse/partner present; for Hukou type, 78.21% were urban, 21.09% were rural, and 0.70% were others.

During the follow-up period, the proportion of samples diagnosed with depression was 32.50%, with the exposed group at 42.65% and the control group at 30.88%. In the exposed group, the depression incidence rate three years after being diagnosed with multimorbidity (2018, Wave 4) was 26.09%, and five years later (2020, Wave 5) was 16.56%.

### 4.3 Multivariate cox analysis

A Cox proportional hazards regression was conducted on all variables after overlap weighting to explore the impact of multimorbidity on the incidence of depression. The results showed that the hazard ratio (HR) was 1.431, with a 95% confidence interval (CI) of 1.202–1.703 and *P* < 0.05. Individuals with multimorbidity had approximately a 43.10% increased risk of developing depression compared to those without multimorbidity.

### 4.4 Sensitivity analysis

Cox proportional hazards regression was performed on the original (unweighted) samples using propensity score matching to validate the findings. The results indicated an HR of 1.415, 95% CI: 1.128–1.775, *P* < 0.05, further supporting the results obtained from overlap weighting. Additionally, to assess the robustness of the identified relationship between multimorbidity and outcomes concerning potential unmeasured confounders, VanderWeele and Ding’s method was used to calculate the E-value, yielding an E-value of 1.88. To render the observed risk ratio (HR = 1.431) no longer significant, unmeasured confounders must be associated with depression and multimorbidity at least 1.88 times the risk. The robustness of the HR obtained from the Cox regression after overlap weighting is moderate.

### 4.5 Subgroup analysis

#### 4.5.1 Grouped by number of multimorbidities.

When the number of multimorbidities was 2, the risk of depression increased relative to those without multimorbidity, with a risk ratio of HR = 1.4239, 95% CI: 1.192–1.701, *P* < 0.05. When the number of multimorbidities was 3, the risk of depression significantly increased, with a risk ratio of HR = 2.0454, 95% CI: 1.503–2.784, *P* < 0.05.

However, when the number of multimorbidities was 4 or more, the risk ratio was HR = 0.8152, 95% CI: 0.444–1.497, *P* > 0.05, indicating that the effect on depression risk was not significant for individuals with 4 or more multimorbidities.

These results indicate that as the number of multimorbidities increases, the risk of depression tends to rise, particularly when there are fewer multimorbidities (such as 2 or 3), where the increase in risk is more pronounced.

#### 4.5.2 Grouped by age.

For individuals younger than 60 years, Cox regression after overlap weighting showed an HR of 1.428, 95% CI: 1.106–1.843, *P* < 0.05, indicating that individuals with multimorbidity had about a 42.80% higher risk of developing depression compared to those without multimorbidity.

For individuals aged 60 years and older, the results indicated an HR of 1.482, 95% CI: 1.221–1.799, *P* < 0.05, suggesting that individuals with multimorbidity had about a 48.20% higher risk of developing depression compared to those without multimorbidity.

The impact of multimorbidity on depression was significant across different age groups, with a greater effect observed in older adults (≥ 60 years).

#### 4.5.3 Grouped by gender.

For the male group, the analysis indicated an HR of 1.341, 95% CI: 1.083–1.659, *P* < 0.05, suggesting that men with multimorbidity had about a 34.10% higher risk of developing depression compared to men without multimorbidity. For the female group, the analysis showed an HR of 1.617, 95% CI: 1.290–2.027, *P* < 0.05, indicating that women with multimorbidity had about a 67.10% higher risk of developing depression compared to women without multimorbidity.

The effect of multimorbidity on depression was significant across different age groups, with a greater impact observed in the female group compared to the male group.

## 5. Discussion

### 5.1 The significant correlation between multiple chronic diseases and depression risk in middle-aged and elderly populations

This study found that individuals with chronic diseases had a significantly increased risk of depression. The following points may explain how multiple chronic diseases contribute to an elevated risk of depression: ① Increased psychological stress and negative emotions. Patients with multiple chronic diseases often experience greater psychological stress and negative emotions [25]. The anxiety, stress, and other emotional challenges brought on by chronic illnesses frequently lead to depressive symptoms. For example, many chronic disease patients feel anxious and stressed due to the long-term management of their illnesses, which in turn affects their emotional state [26]. Furthermore, certain chronic diseases, such as stroke or Alzheimer’s disease, may cause changes in brain function, further exacerbating the risk of depression [27]. Compared to the general population, chronic disease patients typically have complex treatment management needs due to the necessity of long-term treatment, dealing with side effects, and coordinating with multiple healthcare providers, leading to increased psychological burdens and more frequent emotional fluctuations [28–30]. Research has also shown that elderly patients have decreased abilities to recognize and manage emotions, particularly those who struggle to express or identify their feelings, which results in a significantly increased risk of depression [29,30]. ② Long disease course and psychological burden of illness. Chronic disease patients often have a long disease duration accompanied by gradual physical decline, and the long-term health challenges bring continuous psychological burdens that increase the risk of depression. Patients fighting against chronic diseases often experience increased feelings of helplessness, which exacerbates their psychological burden over time. Especially when faced with multiple chronic conditions, the psychological burden becomes even heavier; prolonged illness stress leads to emotional exhaustion, ultimately increasing the risk of depression [31]. For instance, diabetes patients need to manage blood sugar levels and pay attention to diet, exercise, and other lifestyle factors, increasing the burden of self-care. As the disease progresses, these complex lifestyle adjustments and self-management needs may further exacerbate depressive feelings [32]. ③ The association between increased disability, mortality risk, and medical costs. The limitations in physical function, increased mortality risk, and medical cost burdens brought about by multiple chronic diseases create a vicious cycle for the psychological health of chronic disease patients. Studies have shown that with the increase in multiple chronic diseases, the disability rate among patients rises significantly, and the quality of life declines markedly, which typically accompanies an increased risk of depression. Relevant literature also indicates that depressive symptoms not only increase patients’ medical expenses but also lower overall health status, exacerbating their economic and emotional stress [30,33]. Moreover, a meta-analysis supports the positive correlation between multiple chronic diseases and higher mortality risk (HR: 1.44, 95% CI: 1.34–1.55), further backing the notion that multiple chronic diseases may aggravate depressive symptoms [33]. ④ Changes in lifestyle and self-identity. Long-term management of chronic diseases requires patients to make adaptive adjustments in their lifestyles, which can affect their sense of self-identity. For instance, prolonged treatment may lead patients to become distanced from their social circles, and a decline in quality of life may further alter their self-perception, intensifying depressive emotions. For patients with multiple chronic diseases, adverse changes in self-image can increase feelings of loneliness and helplessness, leading to further deterioration of mental health [29].

### 5.2 The relationship between the number of multiple chronic diseases and depression risk in middle-aged and elderly populations

This study found that as the number of multiple chronic diseases increases, the risk of depression rises significantly, especially among patients with 2 to 3 chronic conditions. This trend may be due to the more significant physiological burden and decline in quality of life brought on by multiple chronic diseases, making individuals more susceptible to depression. However, when the number of chronic diseases reaches 4 or more, the increase in depression risk is no longer significant, possibly due to: ① The formation of adaptive mechanisms: Patients may develop adaptive mechanisms or a phenomenon of “psychological numbness” in the long process of coping with multiple chronic diseases, gradually adapting to their multi-morbid status and alleviating the negative impact of emotional stress [34]. ② Enhanced social support: Patients with more multiple chronic diseases often receive more support from family and society, which can provide emotional comfort and practical help, aiding in the alleviation of depressive symptoms [35]. ③ More comprehensive medical support: Patients with 4 or more chronic diseases usually receive more intensive medical and mental health support, further helping to reduce the risk of depression [33]. ④ The non-significant finding in participants with 4 or more chronic conditions may be partly attributable to the small sample size in this subgroup, which limits statistical power.

Among middle-aged and elderly populations, older adults and women have a higher risk of depression. As age increases, older patients face declines in physical function and increasing caregiving demands while managing multiple chronic diseases, leading to a significant rise in depression risk [36]. Additionally, women have a higher risk of depression in the context of multiple chronic diseases, possibly because they are more likely to internalize stress and take on more caregiving responsibilities, making them more susceptible to social and psychological pressures when health deteriorates [37].

The results of this study suggest that mental health management for chronic disease patients should become a focus in public health and clinical practice, particularly for older adults and female patients. Routine chronic disease management should not only include monitoring physiological indicators but also emphasize early screening for depression and psychological support to mitigate the adverse effects of multiple chronic diseases on mental health. Furthermore, the significant association between the increasing number of chronic diseases and depression risk indicates that clinicians should pay special attention to the early recognition of depressive symptoms, especially in patients diagnosed with 2–3 co-morbid chronic diseases.

## 6. Strengths and limitations

This study has several notable strengths. First, it uses high-quality, nationally representative longitudinal data from the China Health and Retirement Longitudinal Study (CHARLS), providing robust evidence on the relationship between multimorbidity and depression in middle-aged and elderly populations. Second, the application of overlap weighting effectively controls for confounding factors, mimicking the balance achieved in randomized controlled trials and enhancing the validity of the results. Third, the use of DAGs to identify key confounders ensures a rigorous methodological approach to confounding adjustment. Additionally, subgroup and sensitivity analyses further validate the robustness of the findings, offering nuanced insights into the impact of multimorbidity across different demographic groups, such as age and gender.

Despite its strengths, this study has several limitations. First, the self-reported nature of chronic disease diagnoses and depressive symptoms may introduce reporting bias or misclassification errors. Second, the study did not include genetic, biochemical, or psychosocial variables that may contribute to depression, which limits the exploration of potential underlying mechanisms. Third, although overlap weighting reduces confounding, residual or unmeasured confounding cannot be fully ruled out. While this approach strengthens internal validity for baseline comparisons, it may overlook the influence of changes in multimorbidity status or other covariates over time. Furthermore, this approach may have created a highly selected healthy control group, potentially biasing the effect estimates and limiting generalizability. Lastly, missing data, particularly for variables like education level and Hukou type, were handled using multiple imputation, but this process may not fully account for biases introduced by incomplete data.

## 7. Conclusion

This study highlights a significant association between multimorbidity and an increased risk of depression in middle-aged and elderly populations in China, with the risk being particularly pronounced among women and older adults. The findings emphasize the compounding mental health burden posed by multimorbidity, especially in individuals with two or three chronic conditions. Interestingly, the diminishing association observed in those with four or more chronic conditions suggests the potential role of adaptive mechanisms, enhanced social support, or comprehensive medical care.

These findings underscore the urgent need for targeted public health interventions to address the mental health challenges faced by individuals with multimorbidity, particularly among vulnerable groups such as older adults and women. Routine chronic disease management should integrate psychological screening and support to mitigate the negative impact of multimorbidity on mental health. Future research should explore the biological and psychosocial mechanisms underlying this relationship and extend these findings to broader populations for greater generalizability.

## Supporting information

S1 Fig Identification of confounding factors using DAGs. (PDF)

## Abbreviations

CHARLS: China Health and Retirement Longitudinal Study
DAGs: Directed Acyclic Graphs
HR: Hazard Ratio
CI: Confidence Interval
CESD-10: 10-item Center for Epidemiological Studies Depression Scale
BMI: Body Mass Index
PSM: Propensity Score Matching
ADL: Activities of Daily Living
IADL: Instrumental Activities of Daily Living
