# Supplementary material for: The impact of multimorbidity on the occurrence of depression among middle-aged and elderly people in China
Source: PLoS One. 2026 Jan 27;21(1):e0340673. doi: 10.1371/journal.pone.0340673 (PMC12843530; doi:10.1371/journal.pone.0340673)
Supplement: S1 Fig — (PDF) [file pone.0340673.s001.pdf]

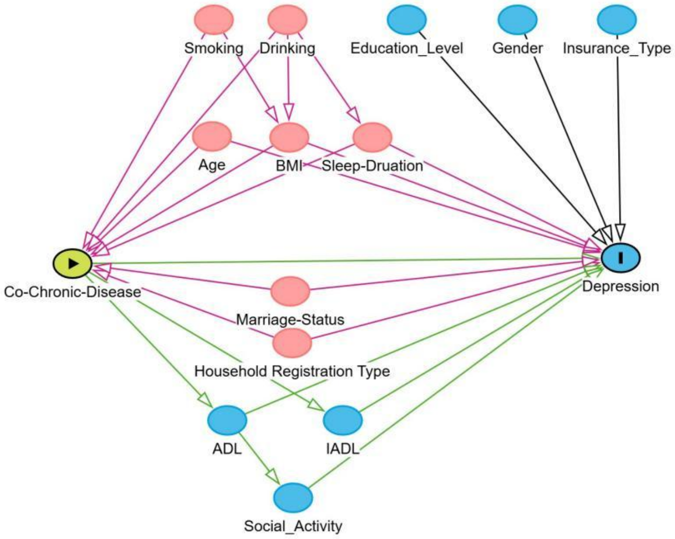

S1 Fig . Identification of Confounding Factors Using DAGs

Note: This figure illustrates the Directed Acyclic Graph (DAG) used in the study to identify potential confounding factors. The variables were selected based on prior literature and empirical evidence. The full DAG used in this study can be accessed at the following link: https://dagitty.net/mdyz2bYq9
